# Development of a new testing protocol to evaluate cooling systems

**DOI:** 10.17179/excli2023-6105

**Published:** 2023-07-06

**Authors:** Miriam Martinez-Albert, Pablo Díaz-García, Eva Bou-Belda

**Affiliations:** 1 Comfort Department, Textile Industry Research Association-AITEX, Alcoy, Spain; 2 Departamento de Ingeniería Textil y Papelera, Universitat Politècnica de València, Alcoy, Spain

**Keywords:** personal cooling systems, sweating manikin, thermal comfort, thermoregulation, heat stress, simulation

## Abstract

Thermal comfort is defined as the user's sensation of thermal well-being. This sensation can be modified by extreme environmental conditions changing the body temperature of the user. Different mechanisms, the thermoregulatory system itself or an external textile system, are required to allow the body to return to their state of well-being. A cooling vest is an example of a smart garment that helps to reduce the user's body temperature in situations of heat stress. There are two different standards to evaluate the performance of this type of clothing: the ASTM F2371-16 standard that uses a thermal manikin and the ASTM F2300-10 standard that uses human subjects for the evaluation. There is a need for simple, objective and affordable tests to evaluate the thermal comfort associated with personal protective equipment use. The aim of this work is to develop a new testing method that combines a thermal manikin with a simulation software and allows to study physiological parameters of a human subject in the body of the thermal manikin.

## Introduction

The standard ISO 7730:2005[18] defines thermal comfort as the thermal sensation experienced by the human being that is related to the overall thermal equilibrium of his body, where such equilibrium, depends on the physical activity, the clothing of the subject and the environmental parameters such as temperature, humidity, air speed and radiant temperature. This thermal equilibrium is obtained when the internal heat production of the human body is equal to its loss to the environment, and it is our body's thermoregulatory system that is responsible for maintaining this thermal neutrality. According to the standard ISO 11079:2007[16], through the appropriate choice of clothing, human beings can control and regulate their body heat depending on the thermal environment in which they find themselves. This standard describes two thermal stress situations in which the individual may find himself due to his clothing: cold stress, when the heat exchange between the individual's body and the environment is too great to maintain the body's thermal balance, and heat stress, when this heat exchange is too small to maintain thermal neutrality. To avoid these heat stress situations, human beings can control and regulate their body temperature depending on the thermal environment by means of appropriate clothing. 

According to the European technical guide CEN/TR 16422:2012[9], a fabric with thermoregulatory properties is a fabric that can influence the thermoregulation of the human body, helping to keep its internal temperature stable and, therefore, keeping the user in a situation of thermal comfort. According to this technical guide, thermal resistance, breathability, air permeability, water penetration resistance, water repellency and moisture transmission are properties that define thermoregulating textiles.

### Thermal manikin 

To test the thermal comfort of ready-made garments, a thermal manikin simulating a person is used in the laboratory. It has different temperature and humidity sensors and a motion system capable of making it walk, thus being able to simulate different activities of the user.

The thermal manikin is designed according to the specifications and requirements of ISO 15831:2004[17] and ASTM F1291:2022[2] standards. According to these standards, the manikin must be made of metal or plastic and constructed to simulate the body of an adult human with anatomically formed head, chest, abdomen, back, buttocks, arms, hands, legs, and feet. The manikin should consist of at least 15 body segments, each independently controlled in relation to surface temperature and heat flow to maintain a constant average body temperature of, e.g. 34.0 ± 0.2 ºC, measured on the surface of all body segments. In these segments, there must be an appropriate temperature sensor to measure this average temperature and a heating system capable of maintaining it.

The thermal manikin allows to measure the thermal insulation (Clo value) and breathability of the garments. This thermal manikin is also used to measure the cooling power of cooling garments using the ASTM F2371:2016[4] standard. This standard analyzes the amount of heat that the personal cooling system can dissipate, but it does not allow to know if the user will be comfortable with the garment or if he/she has a pleasant thermal sensation with it. 

Since more than half a century (Katic et al., 2016[20]) different mathematical models have been developed that allowed simulations to be performed in different environmental conditions with different clothing and activities. Associated with the Thermetrics' Newton thermal manikin, there is a specific software called Manikin PC Software developed by the company ThermoAnalytics Inc. which allows to estimate, apart from other parameters, the thermal comfort that a person would feel with the garment by simulating the thermoregulatory system of the human being in the thermal manikin (Burke et al., 2010[8]). This simulation depends on the environmental conditions in which the manikin is located and allow us to predict subjective parameters of the human body in an objective equipment such as the thermal manikin. The simulation program is based on two mathematical simulation models: the Fiala Model of human thermoregulation (Fiala et al., 1999[12], 2001[13]), and the Berkeley Model of thermal comfort (Huizenga et al., 2000[15]). The Fiala Model is based on a mathematical model to predict the thermal response of the human body in very cold, cold, neutral, warm, hot, and very hot environments. This model predicts skin temperature and core temperature in use conditions based on the following parameters: solar radiation, skin vasodilation, skin vasoconstriction, metabolic activity of the wearer, conduction of the garment, evaporation through the garment, breathing of the wearer, perspiration of the garment, convection of the air surrounding the wearer, hypothalamic thermoregulation and infrared radiation reflected from objects around the wearer. The Berkeley Model allows to calculate the sensation and thermal comfort considering a specific thermal environment and a specific activity based on the ANSI/ASHRAE 55:2020[1] standard.

### Smart textiles: cooling garments

The term smart textile (Van Langenhove et al., 2007[28]) is derived from smart material and is chosen because this type of material adapts to environmental conditions by integrating different functionalities in textile structures. The mechanisms of action of smart textiles are chemical, mechanical, optical, thermal, and electrical. An example of a smart textile is a cooling garment, where a thermal mechanism is used to provide cooling to the wearer in response to an external stimulus, such as an increase in the skin temperature. The first documented cooling garment dates to 1962 and was designed for the aerospace industry (Billingham, 1959[6]).

The study of personal cooling systems is a novel field with very few studies conducted so far, where a growing trend is observed from 2001 onwards, focusing most publications in the last 5 years. In the publications studied, the cooling vest is the most popular garment used as personal cooling system because the trunk area possesses a greater amount of temperature receptors and these are also the most sensitive receptors of the human body (Nishihara et al., 2002[25]). So, it is the part of the body that highly affects the thermal sensation of the user, and the cooling of the trunk causes a drop in core temperature with a rapid spread of this cooling to other areas of the body through the blood (Maruyama and Tamura, 1989[24]).

The internal temperature of a resting person is between 36.1 ºC and 37.1 ºC and the temperature of the hypothalamic thermoregulatory center is the best representative of the internal temperature of the body (Gómez, 2007[14]). In situations of heat or intense activities, this temperature can increase up to 40 ºC, causing the body to suffer hyperthermia, commonly known as heat strain. At that moment, different thermoregulatory mechanisms are activated, such as sweating, vasodilation and hyperventilation. In this situation, the human body also activates its thermoregulatory center which stimulates thirst and the need to dissipate heat. In situations of heat stress, textile materials can help dissipate this body heat, and cooling garments were developed for this purpose. The cooling process of this type of garments has two key points (Bach et al., 2019[5]): the design of the garment to act just when the user needs it and to know what type of heat dissipation is required depending on the use, a passive heat dissipation (evaporation, heat transfer, etc.) or an active heat dissipation (fans, water pumps, etc.). 

There are a multitude of cooling garments on the market, so the user's decision will depend on the cost of the garment, its intended use, its ergonomics, and the thermal sensation of the user. So, the use of cooling garments will depend on objective factors, such as economic and intended use, and subjective factors, such as thermal comfort and ergonomic comfort. 

Cooling garments differ from each other in the mechanism used to dissipate heat. The most common cooling systems in the market are:

#### Cooling mechanism using fluid circulation

These systems use conduction as the heat dissipation mechanism. These garments are fitted with tubes inside which cold water circulates. The fluid is distributed through a pump and cooled usually by one of three mechanisms: compression, absorption, or thermoelectric cooling. Among these mechanisms, it is the thermoelectric system that is the lightest, most resistant, and easiest to use (Bach et al., 2019[5]).

#### Cooling mechanism using body ventilation

Ventilation techniques use convection and evaporation of sweat as a heat dissipation mechanism. In this case, air circulation is forced between the body and the garment (Ren et. al, 2022[27]). These garments have the largest number of published studies, followed by cooling garments with phase change materials (Bach et al., 2019[5]). 

#### Cooling mechanism with phase change materials (PCMs)

These garments have microcapsules with the ability to change from liquid to solid state and vice versa depending on the temperature range in which they work. How effective the PCM is, and the duration of this effect, will depend on the thermal capacity of the PCM, the structure of the fabric and the amount of PCMs applied to the textile (Onder and Sarier, 2015[26]).

#### Other cooling mechanisms

There are other heat dissipation systems such as, for example, personal cooling systems that use water evaporation as a cooling mechanism, systems that use ice to produce cooling, hybrid systems of PCMs in combination with ventilation systems (Lu et al., 2015[23]; Wang et al., 2020[29]; Lai et al., 2017[22]) or a novel system with PCMs that allows the user to ingest ice water (Kim et al., 2020[21]).

### Evaluation methods for cooling garments

There are two standards associated with the analysis of personal cooling systems, the ASTM F2371 standard which is used to determine the cooling power with a thermal manikin and ASTM F2300:2022[3] which evaluates physiological parameters such as skin temperature or internal temperature with users wearing this type of cooling system. There are several studies that question the use of these standards for analyzing the performance of these garments and present alternative test methods. For example, there is a study with users measuring the effectiveness of cooling garments as a function of their wearing time, since the weight of the garment and its ergonomics also play an important role in their thermal comfort (Elson and Eckels, 2015[11]). Other studies use a thermal manikin like the one in the standard, but with modified environmental conditions and a determined test duration (Jetté et al., 2004[19]). There is also a study (Bogerd et al., 2010[7]) where a comparison is made between tests performed with users, the thermal manikin and thermo-physiological simulation systems, concluding that cooling power determined with the ASTM F2371 standard was two times higher compared to the cooling power determined using human participants, while the results obtained with users were very similar to the results obtained using the simulation models. Therefore, there is no consensus regarding the test method used in the analysis of personal cooling systems so the use of ASTM F2371 and ASTM F2300 are two highly questioned test methods: the ASTM F2300 standard provides more physiological information than the ASTM F2371 standard, but the results obtained are more complex to interpret because they involve greater subjectivity by using human users instead of instrumented manikins (Decaens and Vermeersch, 2018[10]). 

The comparison between two analysis methods using the thermal manikin, the standardized test according to ASTM F2371 and an own method developed for this analysis, is presented in this study. 

## Methods

### Materials and instrumentation 

#### Thermal manikin

The manikin used in this study is a Newton thermal manikin from Thermetrics LLC. It is made of a thermally conductive carbon-epoxy resin, fully articulated, composed of 34 body segments. The manikin for this study is equipped with an integrated sweating system consisting of a first layer suit made of elastic fabric and a water pump that distributes water throughout the body of the manikin, thus simulating the sweating system and allowing saturation of the surface of the manikin with water. The body segments to be considered for this study are those covered by the vest and corresponding to the trunk of the manikin: shoulders, lower back, chest, stomach, waist and lumbar region. 

#### Personal cooling system

For these analyses, a cooling vest will be used whose heat dissipation mechanism is by air ventilation. This vest consists of two fans housed in the lumbar region, one on each side, and two other fans housed at the waist, one on each side as well. The vest is connected to an external battery, which, according to the manufacturer, gives it more than 5 hours of autonomy and must be charged before each use. The vest is equipped with a central front zipper and is adjustable on each side by means of two adjustment devices. The total weight of the vest including equipment was 3 kg.

For this study, the appropriate size for the manikin, size L, is used. The specimen, 24 hours prior to each test, is conditioned to the environmental test conditions. 

### Technical procedures

#### Test protocol according to ASTM F2371

In the analysis according to ASTM F2371, the sweat elimination rate and the cooling time provided by the cooling garment are measured, without considering human physiology and obtaining relatively high cooling rates because the manikin's body is completely saturated with water throughout the test and the manikin is not moving, in a static simulation situation. The thermal manikin is located inside a climatic chamber at the environmental conditions established in the standard of 35.0±0.5 ºC ambient temperature, 40±5 % relative humidity and air velocity of 0.4±0.1 m/s. For this test, the manikin is wearing the sweating suit and the sweating system is switched on during the whole test. The body of the manikin according to this standard is heated to a stable body temperature of 35.0±0.5 °C where the energy required to maintain the manikin at this stable temperature is equal to the energy loss of the manikin. Before donning the cooling vest to the manikin, a test under the same environmental conditions is done with the manikin nude, wearing only the sweating suit. 

The ASTM F2371 standard consists of two parts, a previous analysis of the garment without the cooling system operating where the necessary energy to be applied to the manikin to maintain its body temperature at 35.0±0.5 ºC is measured, called PCS baseline test. After this test, a new test is started with the cooling system in operation until the energy needed in this test minus the baseline cooling rate has decreased to 50 W or up to a maximum of 2 hours. For this analysis, we dress the manikin with the vest with the cooling system switched off and perform the baseline test. 

After this initial test, the cooling system is switched on and we carry out the test following the conditions for 2 hours, the maximum time established in this standard that simulates the real use time. Three independent replications are performed, and the average of these replications is calculated. 

#### Test protocol according to the new established testing method 

This new test method developed for cooling garments uses the thermal manikin associated with Manikin PC Software. The thermophysiological data obtained will depend on the environmental conditions of the test, the sample to be analyzed, the activity to be simulated and the duration of the activity. These data will be taken only from the trunk area, the area where the cooling vest will have the greatest influence on the thermal comfort of the user. In this case, the manikin's body temperature is not fixed at 35.0 ºC, but it is regulated by the program itself.

The parameters to be determined in this simulation under the established test conditions are:

- Thermal comfort: it is the comfort predicted by the manikin software presented on a numerical scale. The relationship between the comfort levels given by the manikin and its meaning is presented in Table 1(Tab. 1). It is based on the ASHRAE 7-point scale. 

- Thermal sensation: it is the thermal sensation predicted by the manikin software given on a numerical scale. The thermal sensation levels given by the manikin and its meaning is presented in Table 2(Tab. 2). It is based on to the comfort scale developed by Zhang (2003[30]).

- Skin temperature of the manikin (T_sk_): it is the temperature of the manikin's skin as a function of the climatic conditions used and the simulated activity. 

- Hypothalamus temperature (T_hy_): this is the internal temperature of the manikin as a function of the climatic conditions used and the simulated activity.

The environmental test conditions to be used in this simulation are the same environmental conditions as those established in ASTM F2371 standard. 

Regarding the activity to simulate, we must set in the software the energy that the user would be generating when performing a certain activity in METs, where one MET is defined as the amount of oxygen consumed for a resting activity equivalent to an energy of 58 W/m^2^. Because the cooling garment can be used in different activities, 5 activities have been chosen to be simulated, ranging from very low energy metabolism activities to very high energy metabolism activities. Table 3(Tab. 3) shows the five activities to be simulated and the values associated with metabolic heat production according to ISO 11079. For each activity a duration of 20 min has been set.

Before performing the test on the study sample, the same test is performed without any sample on the manikin, only with the skin body suit, to measure the analysis parameters with the nude manikin and to be able to compare them with those obtained with the vest. This analysis is called nude manikin test. 

Prior to the test of the vest and the test of the nude manikin, it is necessary to have the body of the manikin in thermoneutral conditions. These neutral conditions, in case of the software, are a mean skin temperature of 34.4 ºC and hypothalamus temperature of 37.0 ºC. In this test, we place the climatic chamber at the test conditions with the manikin inside and left to reach its thermoneutrality conditions controlled by the software.

This procedure is performed prior to the test of the nude manikin and prior to the test of the manikin with the cooling vest. 

After the thermoneutral test, in the test of the cooling vest, the cooling system is switched on and the lowest energy activity, 1.4 METs, is programmed with the option of the software called Model Control for 20 min. Every 20 min, the METs will be increased until finally reaching the activity of 6.9 METs with a total testing time of 100 min.

## Results

### Results according to ASTM F2371

**Figure 1:**
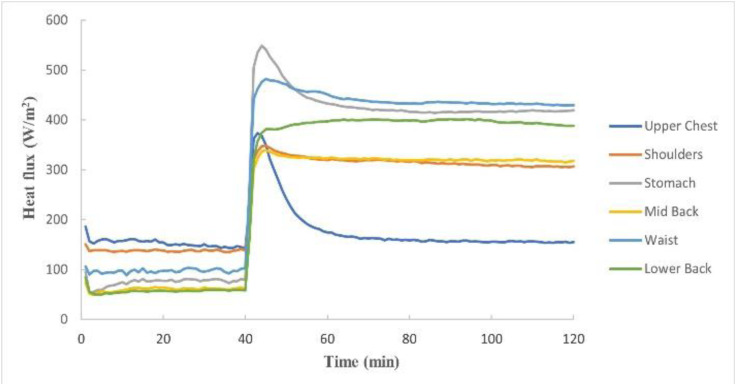
Heat flux of each segment of the trunk through the time for the test according to ASTM F2371

**Figure 2:**
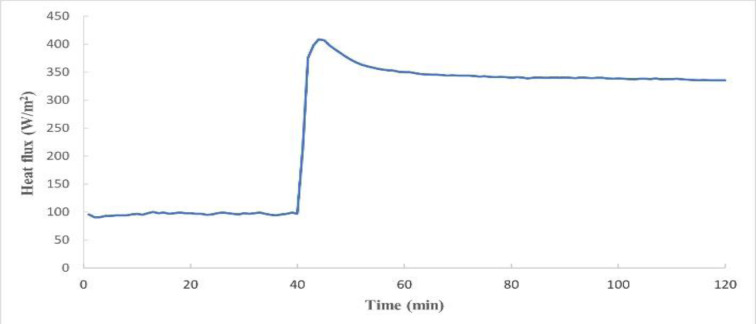
Heat flux of the whole trunk through the time for the test according to ASTM F2371

Figure 1(Fig. 1) shows the average energy required by each body segment of the manikin to maintain its temperature at 35.0±0.5 ºC of the trunk area as a function of the test time and Figure 2(Fig. 2) shows the energy required for the whole trunk zone in total. As can be seen in both figures, the first 40 min of the test are those corresponding to the baseline test, where the manikin was wearing the vest without the cooling system operating and where the last 30 min correspond to the steady state. Once the steady state was reached, the cooling system was switched on, and it was observed for up to 120 min that the energy required by each body segment to maintain the temperature at 35.0±0.5 ºC under sweating conditions was higher than that of the baseline test in all the segments and the entire trunk. This means that when the cooling system was switched on, it lowered the temperature of the manikin segments until it raised again to 35.0±0.5 ºC, requiring a higher energy input to reach this stable temperature.

Regarding the cooling power, an average steady-state energy of 96 W/m^2^ has been obtained without operating the cooling system, this is the baseline cooling power, and when the cooling system was switched on, the average energy required in the trunk area was 348 W/m^2^ and it remained around that value until the end of the test. The corresponding value with the manikin nude was 166 W/m^2^. So, in this case, the air-cooling vest had an effective cooling power of 252 W/m^2 ^comparing on versus off, and an effective cooling power of 182 W/m^2^ comparing vest switched on with the no vest condition. 

### Results according to the new testing method

#### Skin temperature (T_sk_)

As can be seen in Table 4(Tab. 4), the skin of the thermal manikin dressed with the cooling vest for a low intensity activity of 1.4 METs reached a temperature of 34.3 ºC while in the nude test it reached 35.3 ºC. The skin temperature of the manikin with the operating vest increased proportionally to the intensity of the activity until the activity of 6.9 METs, where the skin temperature increased to around 39 ºC for both the nude manikin test and the test with the cooling vest.

#### Hypothalamus temperature (Thy)

Table 5(Tab. 5) shows that in the nude manikin test and the test of the manikin wearing the cooling vest the hypothalamus temperature was 36.9-37.0 ºC with 1.4 METs. 

When the intensity of the activity increased to 2.8 METs, the estimated hypothalamus temperature of the manikin with the operating vest remained constant at around 37 ºC identical to the start of the test, while for the nude manikin the internal temperature reached 38.1 ºC. At a moderate energy metabolism activity of 4 METs, the hypothalamus temperature in both cases became equal at around 38 ºC and increased by 1 ºC as the intensity of the activity increases, reaching about 40 ºC for an activity of 6.9 METs.

#### Thermal comfort level

Table 6(Tab. 6) shows the predicted thermal comfort levels in the whole test. With the nude manikin the comfort level was slightly uncomfortable with a value of -1. On the other hand, in the test of the manikin with the cooling system, the predicted level for an activity of 1.4 and 2.8 METs was comfortable with a value of 2, while at 4 METs the comfort level decreased to 0, neutral comfort, moving to level of -1, slight discomfort, in activities of 5 and 6.9 METs. 

#### Thermal sensation level

The results regarding the thermal sensation level are shown in Table 7(Tab. 7). It was observed that with the manikin without the cooling garment, the estimated thermal sensation was level 2 throughout the test, that is a hot sensation, while for the manikin with the cooling vest in activities of 1.4 and 2.8 METs, a neutral sensation of level 0 was predicted. When the intensity of the activity increased to 4 METs, the sensation was increased to level 1, slightly hot, passing to a level 2, sensation of hot, in high and very high metabolism activities.

## Discussion

Analyzing the results obtained on the manikin with the air-cooling vest in the test according to ASTM F2371 standard, a large difference in energy has been observed between the baseline test and the rest of the test with the cooling system on, because when it is operating, the manikin, at the same sweating conditions, needs more energy to maintain its surface temperature stable as required by the standard. Therefore, due to this difference in energy, it has been found that the cooling vest has a very high and constant effective cooling power during the 2 hours of the test, producing a cooling of the manikin segments almost at the same instant in which the cooling system of the garment is switched on. If we compare the results of the manikin nude with the results of the manikin wearing the vest with the cooling system operating, the heat loss was 86 W/m^2^ higher with the manikin wearing the vest. This means that the body of the manikin is cooler with the cooling vest operating compared to the nude condition. Thus, with this standardized method we have confirmed that the cooling vest is effective. 

As indicated in the introduction, the internal temperature of a resting person is between 36.1 ºC and 37.1 ºC, whereas, in situations of heat or intense activities, this temperature can increase up to 40.0 ºC (Gómez, 2007[14]). These data are supported by the predicted responses of the new testing protocol where in the nude manikin test and in the test with the cooling vest, the hypothalamus temperature started from a temperature of 36.9-37 °C, and increased with the intensity of the manikin's activity, reaching a hypothalamus temperature of 40.1 °C in both cases. Predicted skin temperature also increased with the activity simulated for the manikin, following an expected trend. Looking at the difference between internal temperature and skin temperature in both cases, for low, low-medium and medium energy activities, the difference in temperature for the manikin wearing the cooling vest is 2.3 ºC while the difference in temperature for the nude manikin is 1.8 ºC, so there is 0.5 ºC of difference using the cooling vest or not using it at those activities. At high and very high activities this difference changed by only 0.2 ºC. If we look at the results for a light activity, we find the higher temperature gradient between the skin and the hypothalamus, 1.7 ºC in case of the manikin nude and 2.6 ºC in case of the manikin with the cooling vest, so the core-to-skin temperature gradient increased by 0.9 ºC in case of using the cooling vest. This activity corresponds to the same activity simulated in the standard so, at this activity, the manikin wearing the cooling vest presents 1.0 ºC lower skin temperature than the nude manikin. Regarding the thermal comfort and sensation results of the nude manikin, since the gradient between the skin temperature and the hypothalamus temperature was only 2.1 ºC at maximum, this small difference was associated with a predicted level 2 of sensation, hot, during the whole test and, therefore, a level -1 in comfort, meaning slightly uncomfortable. This sensation of hot and slight discomfort did not change throughout the test because the manikin did not present any type of garment that could dissipate the heat. For the manikin wearing the cooling vest, the thermal sensation improved compared to the nude manikin until at a high and very high energy metabolism activity, its level in sensation equalized. Therefore, considering that the thermal comfort depends on the sensation, this indicates how the use of the cooling vest could improve the thermal comfort for low, low-medium and medium energy activities only.

Comparing the results of the manikin with the cooling vest obtained in the standardized test and those obtained by the test developed for a low energy activity, the high cooling power of the vest according to the ASTM F2371 coincides with the results obtained in the test developed in this study for the same activity, where the manikin presented a lower skin temperature than the nude manikin with a level 0 of sensation, indicating a neutral thermal sensation, and a level 2 of comfort meaning a comfortable thermal situation. Thus, it is possible to use the testing protocol of this study to give additional subjective information in terms of thermal sensation and thermal comfort about this type of garment. 

### Limitations and future considerations

The present study is not without its limitations that should be considered in the future. Regarding the results obtained with the cooling vest, it has been observed with our own testing method that in high and very high metabolic intensity activities its use is no longer effective, since both, its predicted sensation and thermal comfort, are equal to that predicted for the manikin without any type of garment. In addition, looking at the skin and hypothalamus temperatures, in high and very high activities, these temperatures do not match with the level of comfort of -1 that means slightly uncomfortable. These inconsistent results suggest that the new testing method cannot be applied to high and very high activities, but it can be applied to a light activity, which is the activity simulated in the ASTM F2371 standard.

Although the software was validated with human subjects before its commercialization (Burke et al., 2010[8]), the purpose for a future study is to perform the same in-house testing protocol with human subjects to validate the results of our simulation tests.

## Conclusions

The intention of our study was to compare the performance of a cooling vest with two different methods, one standardized method and another testing protocol developed for this study in order to verify that both test methods can be complementary. The test according to ASTM F2371 calculates the cooling performance of a cooling garment with a thermal manikin in a static position, which is a light activity. The testing method developed in this study uses different activities to simulate physiological parameters of the human body. With the simulation test developed for this study, it has been found that in low energy load activities, the new method gives estimates of objective and interpretable data, such as internal temperature and skin temperature, and additionally predicts subjective data such as thermal sensation and comfort of the user. In summary, it can be affirmed that for the analysis of cooling garments, at the light activity of the standard method, the new method that combines the thermal manikin with the simulation software Manikin PC Software is a fast and simple method of analysis that gives us estimations of both objective and subjective data of the users wearing this type of garments in an objective laboratory equipment. Complementary to the ASTM F2371 standard, simulation methods employing a thermal manikin can be a fast, simple, reliable and cheaper test method, with the potential to replace the tests carried out with subjects. Such methods could be incorporated in a future standard, which should only include simulation methods with scrutinized validity for cooling garment use conditions.

## Acknowledgement

Parts of the content of this paper were presented in the session 'Digital Human Models and Virtual Ergonomics of PPE Systems' at the 10^th^ European Conference on Protective Clothing, 9-12 May, in Arnhem, The Netherlands.

## Figures and Tables

**Table 1:**
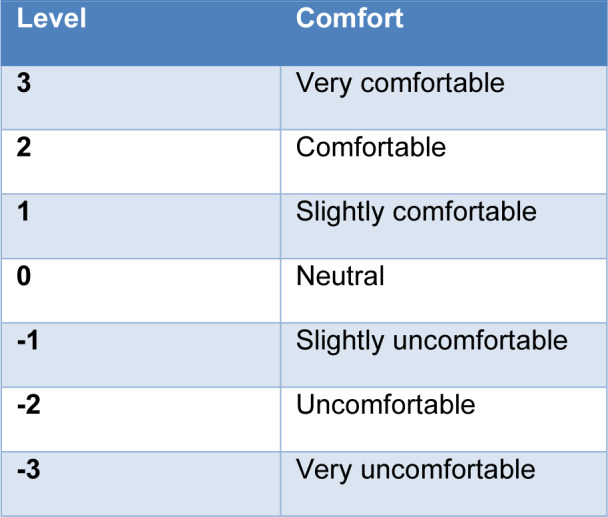
Thermal comfort levels

**Table 2:**
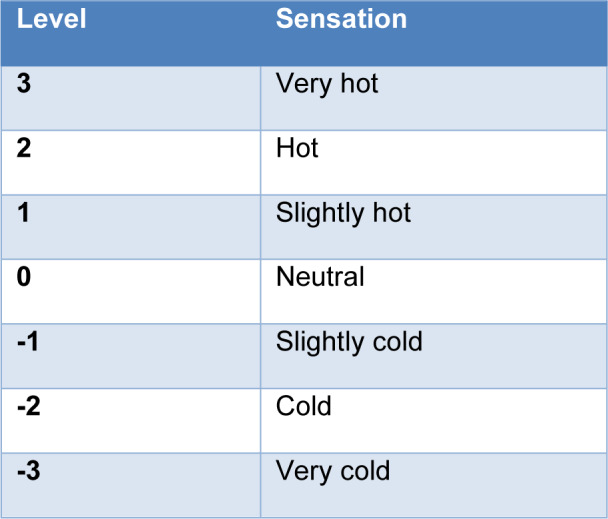
Thermal sensation levels

**Table 3:**
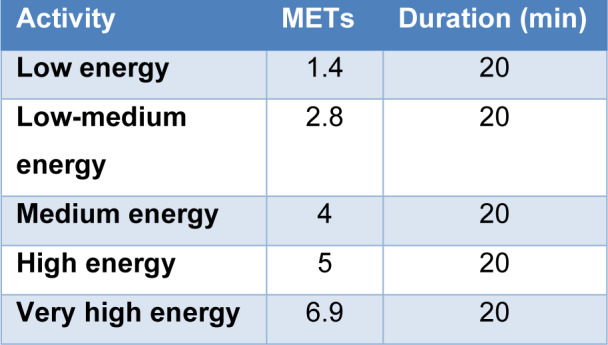
Activities and duration

**Table 4:**
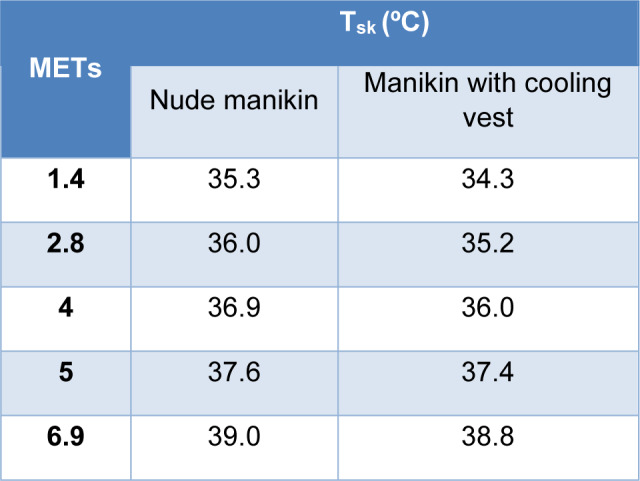
Predicted skin temperature (T_sk_) according to the activity simulated

**Table 5:**
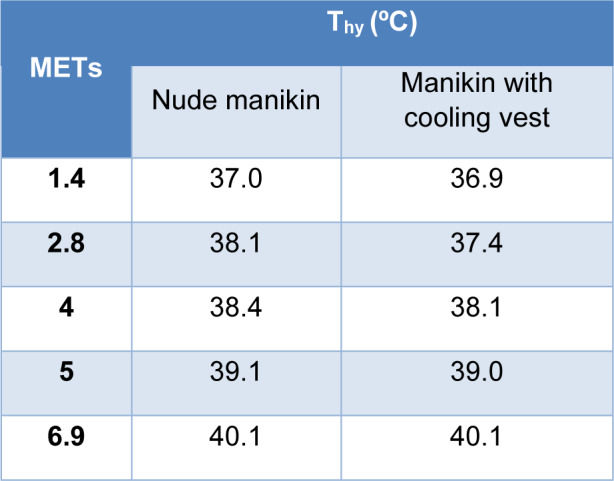
Predicted hypothalamus temperature (T_hy_) according to the activity simulated

**Table 6:**
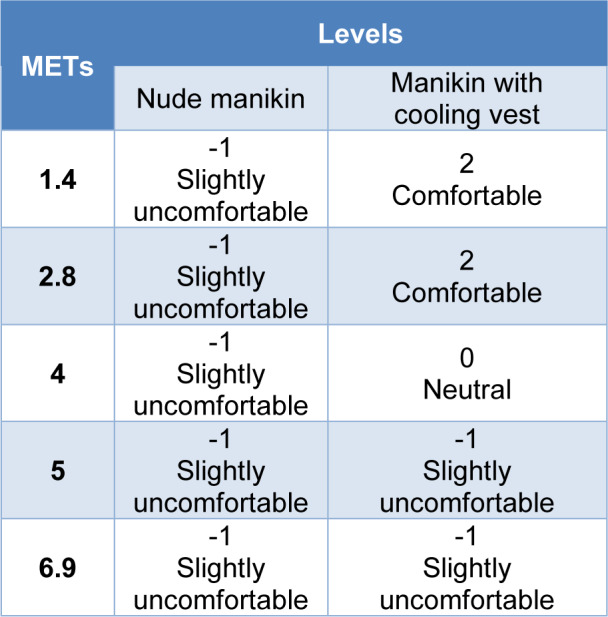
Predicted thermal comfort levels according to the activity simulated

**Table 7:**
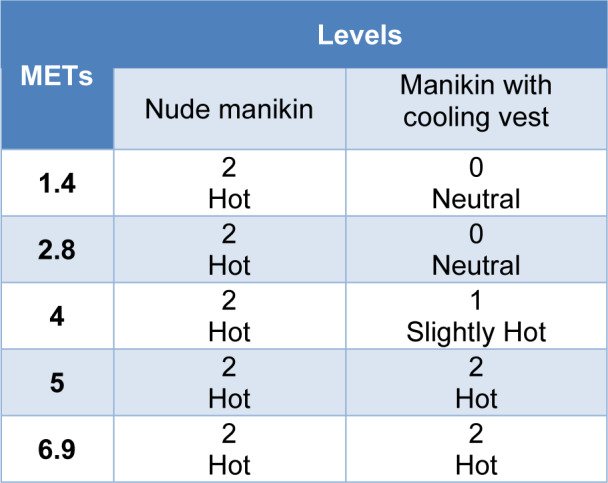
Predicted thermal sensation levels according to the activity simulated
